# Dataset of development of stochastic groundwater flow under uncertain recharge

**DOI:** 10.1016/j.mex.2020.100907

**Published:** 2020-04-30

**Authors:** Sophia Rwanga, Julius Ndambuki

**Affiliations:** a Vaal University of Technology, Department of Civil Engineering, South Africa; b Department of Civil Engineering, Tshwane University of Technology, South Africa

**Keywords:** Development, Groundwater flow, Recharge, Stochastic, Uncertain, MODFLOW Software

## Abstract

Groundwater is a valuable resource of limited extent both in quantity and in space. In order to ensure its careful use, proper evaluation of its availability is required. The majority of groundwater flow numerical models still provide only deterministic predictions with no supplementary information on uncertainty predictions. Stochastic groundwater management aims at treating uncertainty within decision oriented models in a logical and systematic manner which cannot be experienced in deterministic modelling approach. This paper presents a detailed methodology followed in the development of a stochastic groundwater model under uncertain recharge. The methodology is comprised of two steps (1) The development of a groundwater flow model and (2) the development of a stochastic solution- stochastic groundwater flow model considering recharge as uncertain parameter.

• The groundwater flow model was developed and simulated using MODFLOW 2000 software and calibrated using Parameter Estimation Program (PEST). Validated model compared well with calibrated model.
• The uncertainty in recharge was propagated to the flow model through Monte Carlo (MC) sampling technique.
• The methodology demonstrated the importance of developing groundwater flow models that acknowledge the existence of recharge uncertainty and hence consider it in the development of groundwater management solutions.

## Highlights

• The methodology presented in this paper will be useful for groundwater researchers and modelers.

## Graphical abstract

**Figure:**
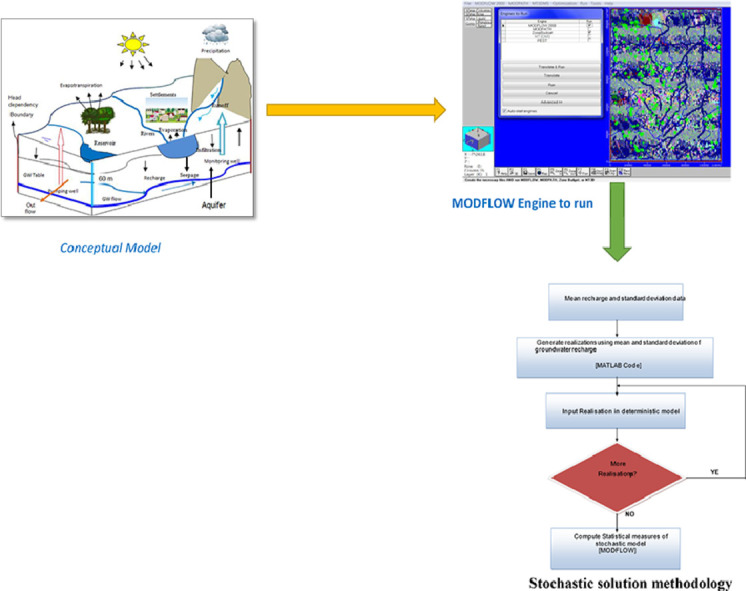

Specifications table

**Table:** 

Subject Area	Engineering
More specific subject area:	Water - Civil Engineering
Method name:	*Groundwater Modelling*
Name and reference of original method:	Steady - state Numerical model was developed using MODFLOW – 2000 [3]. The governing partial differential equation for a confined aquifer used in MODFLOW is;
	$S_{s}=\frac{\text{dh}\;}{\text{dt}}=\frac{d}{\text{dx}\;}\left(K_{x}\frac{\text{dh}}{\text{dx}}\right)+\frac{d}{\text{dy}}\left(K_{y}\frac{\text{dh}}{\text{dy}}\right)+\frac{d}{\text{dz}}\left(K_{z}\frac{\text{dh}}{\text{dz}}\right)\pm W$
	MODFLOW is a computer program that numerically solves the three – dimensional groundwater flow equation for a porous medium by using Finite difference method [3].The code is based on the flow equation of Darcy and mass continuity equation. In this study the groundwater flow was estimated using groundwater flow equation.
How data were acquired:	Data was collected from different sectors listed below; - Department of water affairs (DWAF) – Wells data - National Groundwater Archive (NGA) – Wells data - South African weather services (SAWS) – Climatic data - Consultant's offices e.g (GRIP, S&W Limpopo, VSA, GPM and APHANE – CONSULTING) - Boreholes, Aquifers properties data - Geosciences department – Geological shapefiles, Landuse information
Data format:	Raw
Software used for data analysis:	ArcGIS and Microsoft Excel
Software used for simulation:	MODFLOW 2000
Software used for programme coding:	MATLAB 2004a
Data accessibility:	Data represented with the article
Description of borehole data collection:	Site was identified, site visit done for some of the boreholes and their geographic coordinates were recorded using a handheld global positioning system (GPS) device.
Value of the data:	• Generation of recharge field for stochastic analysis • The development of a distributed groundwater flow model for the study area aimed at providing information on the groundwater aquifer system as well as the water budget associated with it. • It also helped in understanding the behaviour of the groundwater system in response to stress due to groundwater abstractions. • The developed model can be used for management of groundwater through scenarios analysis and which will aid in decision making.

## 1 Methods details

### 1.1 Methodology on the development of a groundwater flow model

Development of steady – state, numerical model was done using [3] MODFLOW – 2000 [3]. The model was discretized into 117 columns and 164 rows. The grid cell size was 1000 m in both the x and y- directions. Due to insufficient log data, a single model of confined aquifer layer 60 m thickness (obtained from Groundwater Resource Information Project (GRIP) dataset) was used to simulate flow and mass balance of the study area. The modelled domain covered an area of 19188 km^2^ which was projected with UTM projection system. Fig. 1a represent the discretization of the modelled area. During model development, the following data were used as input in MODFLOW;

***Elevation:*** The elevation data was gathered from elevation map (Fig. 1b) processed in ArcGIS. After importing the elevation, the model executed interpolation of the imported data through Neighbouring Interpolation technique.

**Wells**

Boreholes data was obtained from GRIP Limpopo and National Groundwater Archive (NGA). During this process, two types of wells data was prepared; (i) pumping wells and (ii) observation wells. A total of 203 boreholes were captured on the model (Table 1) with only 100 randomly selected dataset of pumping wells shown in the Table 1 for clarity. Fig. 1c shows the distribution of pumping wells in the study area. Observation wells were added in the model for the purpose of model calibration only. A total of 30 observation wells were used for this task (Table 2). The choice of observation wells was based on (i) recent data availability (ii) completeness of the data and (iii) spatial distribution of the wells. Data processing for wells was done through ArcGIS using extraction and clipping on Geoprocessing tool and later imported in MODFLOW through the import function.

**Table 1:** Pumping wells dataset used in MODFLOW model.

Well	X-model	Y-model	Start	Stop	Rate[m^3/d]	Screen	Screen
Name	coor[m]	coord[m]	Time[day]	Time[day]		Top1[m]	Bot1[m]
PW1	4.62E+03	9.51E+04	0.00E+00	3.60E+02	-2.64E+02	8.74E+02	8.73E+02
PW10	1.01E+04	5.40E+04	0.00E+00	3.60E+02	-1.60E+02	9.58E+02	9.56E+02
PW100	4.69E+04	5.13E+04	0.00E+00	3.60E+02	-3.24E+02	1.21E+03	1.21E+03
PW101	6.27E+04	4.79E+04	0.00E+00	3.60E+02	-1.94E+02	1.29E+03	1.29E+03
PW102	7.27E+04	4.87E+04	0.00E+00	3.60E+02	-2.42E+02	1.23E+03	1.22E+03
PW103	7.63E+04	4.29E+04	0.00E+00	3.60E+02	-1.56E+02	1.24E+03	1.24E+03
PW104	5.62E+04	8.44E+04	0.00E+00	3.60E+02	-8.60E+03	1.13E+03	1.13E+03
PW105	5.69E+04	8.40E+04	0.00E+00	3.60E+02	-2.07E+03	1.14E+03	1.13E+03
PW106	5.72E+04	8.71E+04	0.00E+00	3.60E+02	-1.95E+03	1.09E+03	1.09E+03
PW107	6.33E+04	8.28E+04	0.00E+00	3.60E+02	-1.99E+02	1.11E+03	1.11E+03
PW108	6.34E+04	8.30E+04	0.00E+00	3.60E+02	-2.03E+02	1.13E+03	1.13E+03
PW109	4.51E+04	6.29E+04	0.00E+00	3.60E+02	-1.56E+02	1.16E+03	1.16E+03
PW11	7.63E+03	4.28E+04	0.00E+00	3.60E+02	-1.76E+03	9.60E+02	9.59E+02
PW111	4.87E+04	6.12E+04	0.00E+00	3.60E+02	-4.54E+02	1.19E+03	1.19E+03
PW112	6.01E+04	5.72E+04	0.00E+00	3.60E+02	-2.16E+03	1.25E+03	1.25E+03
PW113	4.32E+04	4.66E+04	0.00E+00	3.60E+02	-5.01E+02	1.25E+03	1.24E+03
PW114	4.28E+04	5.42E+04	0.00E+00	3.60E+02	-4.32E+02	1.18E+03	1.18E+03
PW116	6.98E+04	5.25E+04	0.00E+00	3.60E+02	-7.95E+02	1.23E+03	1.23E+03
PW117	6.45E+03	1.13E+05	0.00E+00	3.60E+02	-1.08E+02	8.23E+02	8.21E+02
PW118	7.24E+03	1.36E+05	0.00E+00	3.60E+02	-1.47E+02	7.83E+02	7.82E+02
PW119	6.67E+03	1.24E+05	0.00E+00	3.60E+02	-2.38E+02	8.30E+02	8.29E+02
PW120	5.65E+03	1.24E+05	0.00E+00	3.60E+02	-2.33E+02	8.16E+02	8.14E+02
PW121	7.36E+03	1.25E+05	0.00E+00	3.60E+02	-1.81E+02	8.09E+02	8.07E+02
PW122	6.21E+03	1.12E+05	0.00E+00	3.60E+02	-9.50E+02	8.33E+02	8.31E+02
PW123	7.46E+03	1.25E+05	0.00E+00	3.60E+02	-5.75E+02	8.13E+02	8.11E+02
PW124	1.97E+04	1.24E+05	0.00E+00	3.60E+02	-2.38E+02	9.23E+02	9.21E+02
PW125	3.42E+04	1.17E+05	0.00E+00	3.60E+02	-5.27E+02	9.25E+02	9.24E+02
PW126	2.25E+04	1.17E+05	0.00E+00	3.60E+02	-3.46E+02	1.01E+03	1.01E+03
PW129	2.95E+04	1.34E+05	0.00E+00	3.60E+02	-2.16E+02	9.28E+02	9.27E+02
PW13	1.61E+04	2.78E+04	0.00E+00	3.60E+02	-9.12E+02	9.82E+02	9.81E+02
PW130	1.51E+04	1.31E+05	0.00E+00	3.60E+02	-1.47E+02	8.66E+02	8.65E+02
PW131	3.53E+04	1.13E+05	0.00E+00	3.60E+02	-4.97E+02	9.54E+02	9.52E+02
PW132	3.49E+04	1.12E+05	0.00E+00	3.60E+02	-1.68E+02	9.49E+02	9.48E+02
PW134	2.72E+04	1.15E+05	0.00E+00	3.60E+02	-3.07E+02	9.44E+02	9.43E+02
PW135	2.98E+04	1.22E+05	0.00E+00	3.60E+02	-2.76E+02	9.87E+02	9.86E+02
PW136	1.40E+04	1.37E+05	0.00E+00	3.60E+02	-6.91E+01	7.99E+02	7.98E+02
PW137	1.79E+04	1.24E+05	0.00E+00	3.60E+02	-1.38E+03	9.07E+02	9.06E+02
PW138	1.64E+04	1.10E+05	0.00E+00	3.60E+02	-1.86E+02	9.60E+02	9.58E+02
PW139	1.61E+04	1.10E+05	0.00E+00	3.60E+02	-7.78E+01	9.45E+02	9.43E+02
PW14	1.54E+04	2.90E+04	0.00E+00	3.60E+02	-2.20E+02	9.81E+02	9.80E+02
PW140	3.95E+04	1.18E+05	0.00E+00	3.60E+02	-5.01E+02	9.02E+02	9.00E+02
PW141	3.94E+04	1.18E+05	0.00E+00	3.60E+02	-1.30E+02	9.07E+02	9.06E+02
PW142	3.78E+04	1.36E+05	0.00E+00	3.60E+02	-5.18E+01	9.09E+02	9.08E+02
PW143	5.38E+04	1.19E+05	0.00E+00	3.60E+02	-2.38E+03	8.83E+02	8.82E+02
PW144	5.00E+04	1.18E+05	0.00E+00	3.60E+02	-2.16E+02	8.72E+02	8.70E+02
PW145	5.25E+04	1.20E+05	0.00E+00	3.60E+02	-4.75E+02	8.64E+02	8.62E+02
PW146	4.15E+04	1.11E+05	0.00E+00	3.60E+02	-2.16E+02	9.31E+02	9.30E+02
PW147	3.85E+04	1.21E+05	0.00E+00	3.60E+02	-2.59E+01	9.08E+02	9.06E+02
PW149	4.50E+04	9.48E+04	0.00E+00	3.60E+02	-3.28E+03	1.05E+03	1.05E+03
PW15	1.57E+04	2.78E+04	0.00E+00	3.60E+02	-9.94E+01	9.87E+02	9.86E+02
PW150	5.05E+04	1.06E+05	0.00E+00	3.60E+02	-1.79E+03	9.65E+02	9.63E+02
PW151	6.21E+04	1.03E+05	0.00E+00	3.60E+02	-3.39E+03	9.64E+02	9.62E+02
PW152	4.98E+04	1.08E+05	0.00E+00	3.60E+02	-8.39E+03	9.43E+02	9.41E+02
PW153	4.35E+04	1.02E+05	0.00E+00	3.60E+02	-1.73E+02	9.83E+02	9.81E+02
PW154	4.36E+04	1.01E+05	0.00E+00	3.60E+02	-4.88E+02	9.93E+02	9.91E+02
PW155	4.66E+04	9.82E+04	0.00E+00	3.60E+02	-4.92E+03	1.02E+03	1.02E+03
PW156	4.62E+04	9.75E+04	0.00E+00	3.60E+02	-1.03E+04	1.01E+03	1.01E+03
PW157	4.69E+04	9.87E+04	0.00E+00	3.60E+02	-5.07E+03	1.02E+03	1.02E+03
PW158	5.33E+04	9.35E+04	0.00E+00	3.60E+02	-4.65E+03	1.03E+03	1.03E+03
PW159	5.13E+04	1.06E+05	0.00E+00	3.60E+02	-2.59E+01	9.59E+02	9.58E+02
PW16	1.91E+04	2.83E+04	0.00E+00	3.60E+02	-2.16E+02	1.00E+03	1.00E+03
PW160	4.62E+04	9.80E+04	0.00E+00	3.60E+02	-4.23E+03	1.02E+03	1.02E+03
PW161	4.56E+04	9.79E+04	0.00E+00	3.60E+02	-1.06E+04	1.02E+03	1.02E+03
PW162	3.80E+04	1.05E+05	0.00E+00	3.60E+02	-3.97E+02	9.68E+02	9.67E+02
PW163	4.38E+04	1.05E+05	0.00E+00	3.60E+02	-8.64E+01	9.67E+02	9.65E+02
PW164	5.44E+04	1.03E+05	0.00E+00	3.60E+02	-3.46E+02	9.75E+02	9.73E+02
PW165	4.07E+04	1.00E+05	0.00E+00	3.60E+02	-1.81E+02	1.00E+03	1.00E+03
PW166	6.04E+04	1.02E+05	0.00E+00	3.60E+02	-9.59E+02	9.75E+02	9.74E+02
PW167	6.36E+04	1.06E+05	0.00E+00	3.60E+02	-4.53E+03	9.80E+02	9.79E+02
PW168	6.30E+04	9.97E+04	0.00E+00	3.60E+02	-6.53E+03	1.03E+03	1.03E+03
PW169	6.35E+04	1.01E+05	0.00E+00	3.60E+02	-7.47E+03	9.57E+02	9.55E+02
PW17	3.22E+04	3.09E+04	0.00E+00	3.60E+02	-9.50E+02	1.17E+03	1.17E+03
PW170	6.32E+04	9.99E+04	0.00E+00	3.60E+02	-6.39E+02	9.91E+02	9.90E+02
PW171	6.30E+04	1.00E+05	0.00E+00	3.60E+02	-2.33E+02	1.01E+03	1.01E+03
PW172	6.37E+04	1.02E+05	0.00E+00	3.60E+02	-4.75E+02	9.96E+02	9.95E+02
PW173	7.04E+04	9.94E+04	0.00E+00	3.60E+02	-4.32E+01	9.93E+02	9.91E+02
PW174	6.29E+03	1.10E+05	0.00E+00	3.60E+02	-9.12E+02	8.30E+02	8.29E+02
PW175	7.34E+04	8.18E+04	0.00E+00	3.60E+02	-5.83E+02	1.07E+03	1.07E+03
PW176	1.11E+05	8.38E+04	0.00E+00	3.60E+02	-1.21E+02	1.06E+03	1.06E+03
PW177	1.11E+05	8.39E+04	0.00E+00	3.60E+02	-8.13E+03	1.06E+03	1.06E+03
PW178	1.10E+05	8.32E+04	0.00E+00	3.60E+02	-1.08E+02	1.08E+03	1.08E+03
PW179	1.10E+05	8.17E+04	0.00E+00	3.60E+02	-2.59E+01	1.10E+03	1.10E+03
PW18	3.28E+04	3.07E+04	0.00E+00	3.60E+02	-5.40E+02	1.18E+03	1.18E+03
PW180	1.12E+05	8.88E+04	0.00E+00	3.60E+02	-4.32E+02	1.02E+03	1.02E+03
PW181	1.01E+05	8.30E+04	0.00E+00	3.60E+02	-3.07E+02	1.01E+03	1.01E+03
PW182	1.03E+05	8.30E+04	0.00E+00	3.60E+02	-2.76E+02	1.01E+03	1.01E+03
PW183	1.13E+05	8.25E+04	0.00E+00	3.60E+02	-1.51E+02	1.13E+03	1.13E+03
PW184	1.12E+05	8.60E+04	0.00E+00	3.60E+02	-6.70E+02	9.80E+02	9.78E+02
PW185	1.11E+05	8.53E+04	0.00E+00	3.60E+02	-3.07E+02	1.04E+03	1.04E+03
PW188	6.94E+04	3.21E+04	0.00E+00	3.60E+02	-1.38E+02	1.33E+03	1.33E+03
PW189	8.13E+04	4.61E+04	0.00E+00	3.60E+02	-7.56E+03	1.18E+03	1.18E+03
PW19	3.39E+04	2.95E+04	0.00E+00	3.60E+02	-9.07E+01	1.19E+03	1.19E+03
PW190	8.12E+04	4.58E+04	0.00E+00	3.60E+02	-1.08E+04	1.18E+03	1.18E+03
PW191	8.14E+04	4.55E+04	0.00E+00	3.60E+02	-1.43E+03	1.18E+03	1.17E+03
PW193	1.03E+05	7.97E+04	0.00E+00	3.60E+02	-1.06E+03	1.03E+03	1.03E+03
PW194	1.02E+05	8.08E+04	0.00E+00	3.60E+02	-1.14E+03	1.02E+03	1.02E+03
PW195	1.06E+05	4.82E+04	0.00E+00	3.60E+02	-6.48E+02	1.15E+03	1.15E+03
PW196	1.14E+05	5.81E+04	0.00E+00	3.60E+02	-2.16E+02	1.25E+03	1.25E+03
PW197	1.14E+05	5.95E+04	0.00E+00	3.60E+02	-1.83E+03	1.20E+03	1.20E+03
PW199	1.15E+05	5.39E+04	0.00E+00	3.60E+02	-6.48E+01	1.30E+03	1.30E+03
PW20	3.45E+04	3.06E+04	0.00E+00	3.60E+02	-1.08E+02	1.19E+03	1.19E+03

**Fig. 1a:**
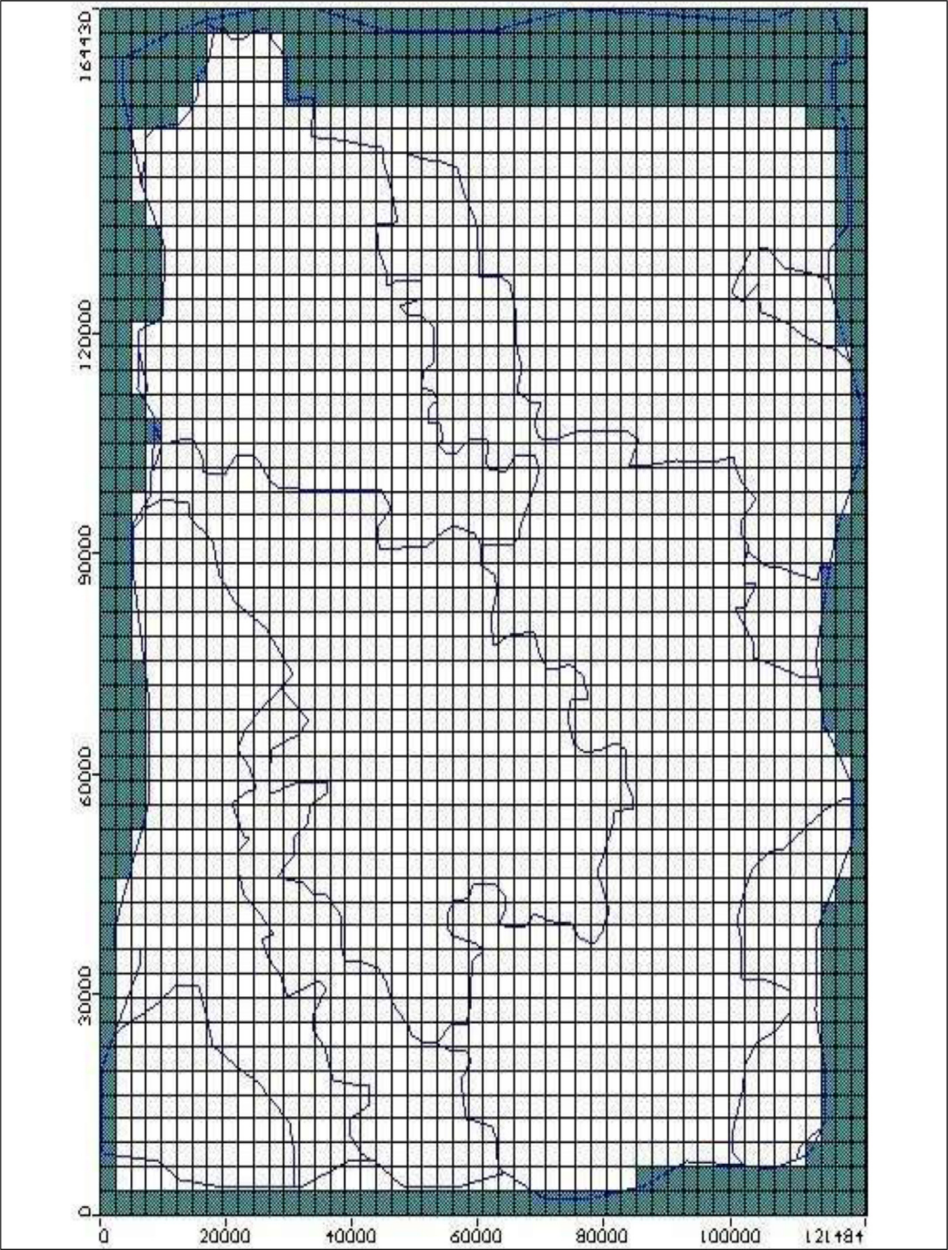
Discretization of the modelled area.

**Fig. 1b:**
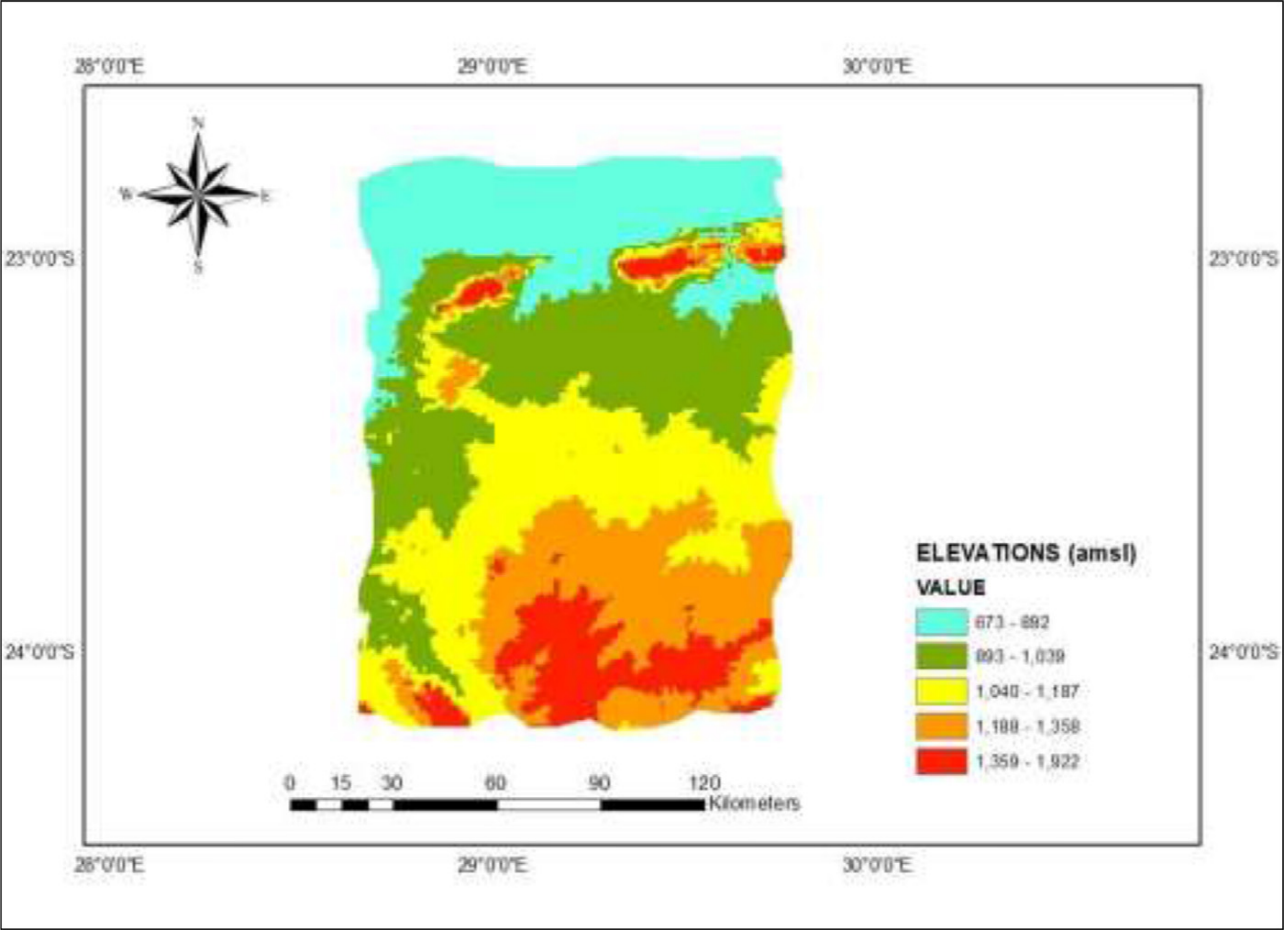
Elevation map.

**Fig. 1c:**
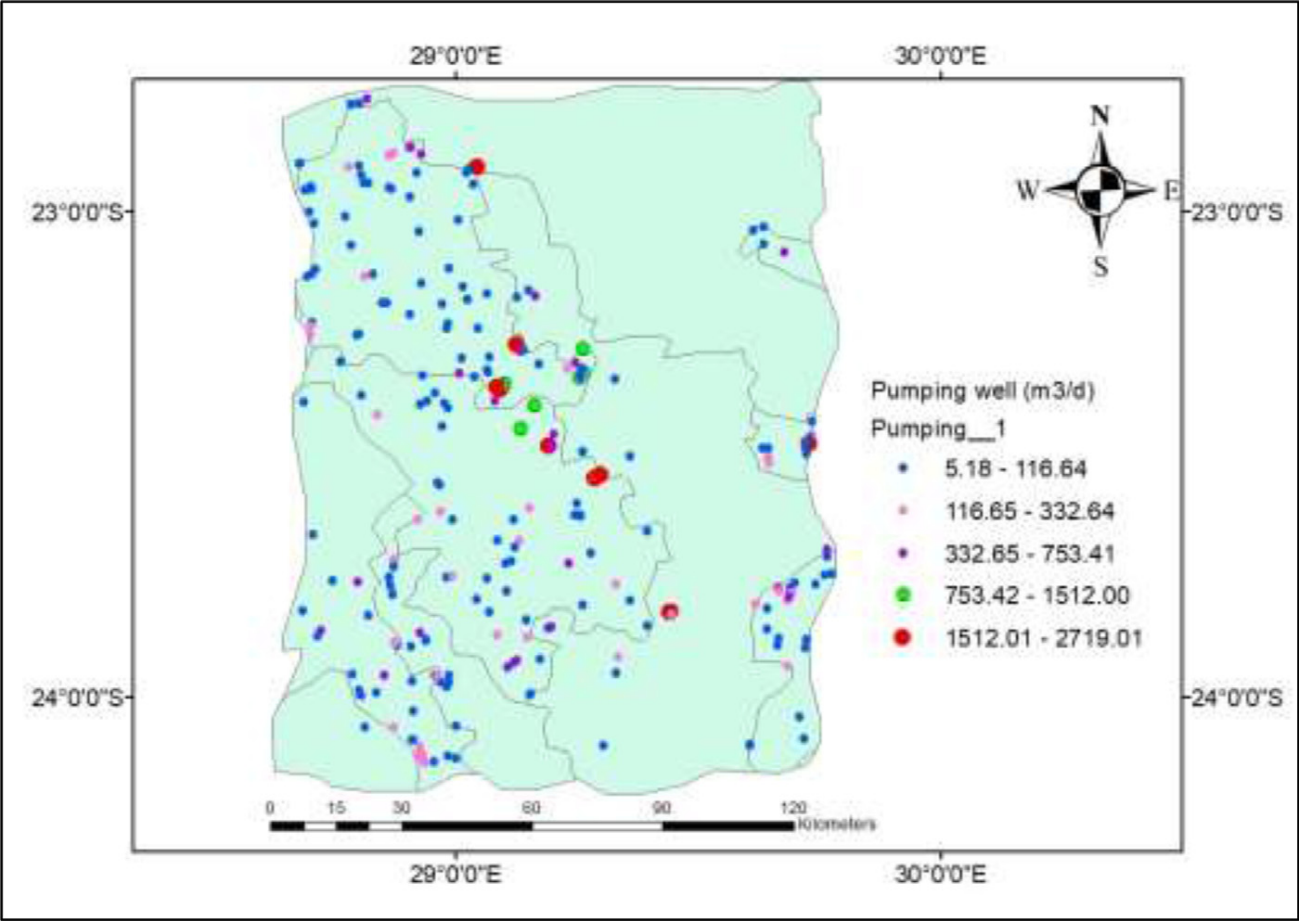
Pumping wells distribution and their pumping rates.

**Table 2:** Observation wells used in Calibration procedure.

Well Name	X [m]	Y [m]	Elevation[m]	Screen ID	Screen Elev. [m]	Obs. Time [day]	HEAD [m]
OBW1	701679	7333460	1231.38	OB1	1109.1	360	1124.1
OBW2	701425	7333910	1231.38	OB2	1108.17	360	1123.17
OBW3	700755	7334390	1221.48	OB3	1103.96	360	1118.96
OBW4	699738	7335980	1202.49	OB4	1102.52	360	1117.52
OBW5	699392	7336130	1202.49	OB5	1096.76	360	1111.76
OBW6	699015	7336420	1191.99	OB6	1094.89	360	1109.89
OBW7	702462	7332400	1238.65	OB7	1114.38	360	1129.38
OBW8	702058	7333090	1227.61	OB8	1114.62	360	1129.62
OBW9	701957	7332940	1227.61	OB9	1110.34	360	1125.34
OBW10	701865	7332840	1231.38	OB10	1113.49	360	1128.49
OBW11	702211	7332590	1238.65	OB11	1117.65	360	1132.65
OBW12	701899	7332580	1231.38	OB12	1106.79	360	1121.79
OBW13	700549	7433910	1213.25	OB13	906.80	360	921.800
OBW14	681608	7438820	1070.15	OB14	881.80	360	896.800
OBW15	700523	7433970	1213.25	OB15	912.40	360	927.400
OBW16	713390	7414850	1290.51	OB16	1011.73	360	1026.73
OBW17	732503	7414420	1191.58	OB17	1006.8	360	1021.8
OBW18	749642	7356850	1243.8	OB18	1243.8	360	1258.8
OBW19	747207	7356060	1206.8	OB19	1206.8	360	1221.8
OBW20	750941	7350730	1246.8	OB20	1246.8	360	1261.8
OBW21	749616	7355880	1216.8	OB21	1216.8	360	1231.8
OBW22	749742	7352160	1241.8	OB22	1241.8	360	1256.8
OBW23	745289	7363540	1191.8	OB23	1191.8	360	1206.8
OBW24	749420	7364880	1171.8	OB24	1171.8	360	1186.8
OBW25	763775	7346010	1291.8	OB25	1291.8	360	1306.8
OBW26	772557	7369760	1166.8	OB26	1166.8	360	1181.8
OBW27	709298	7460550	1293.74	OB27	826.80	360	841.8
OBW28	706128	7326200	1277.03	OB28	1139.07	360	1154.07
OBW29	706681	7333200	1280.06	OB29	1137.87	360	1152.87
OBW30	701095	7334120	1221.48	OB30	1102.78	360	1117.78

***Aquifer hydraulic conductivities:*** In MODFLOW, the hydrogeology properties of the aquifer use the following: (i) Conductivity (K_x_, K_y_, K_z_) (ii) Storage (S_y_, S_s_,P_eff_,P_tot_) (iii) Initial head and vadoze zone. The groundwater flow model requires property values of conductivity, storage and initial heads for each grid cell in order to run a flow simulation. The parameters of hydraulic conductivity, transmissivity and storage coefficient were obtained from the results of pumping test analysis. Hydraulic conductivity varied between 3.568 × 10^−3^ to 30.78 m/d. The hydraulic conductivities were assigned in five distinct zones based on their geological formation and point hydraulic conductivities (Fig. 1d).

**Fig. 1d:**
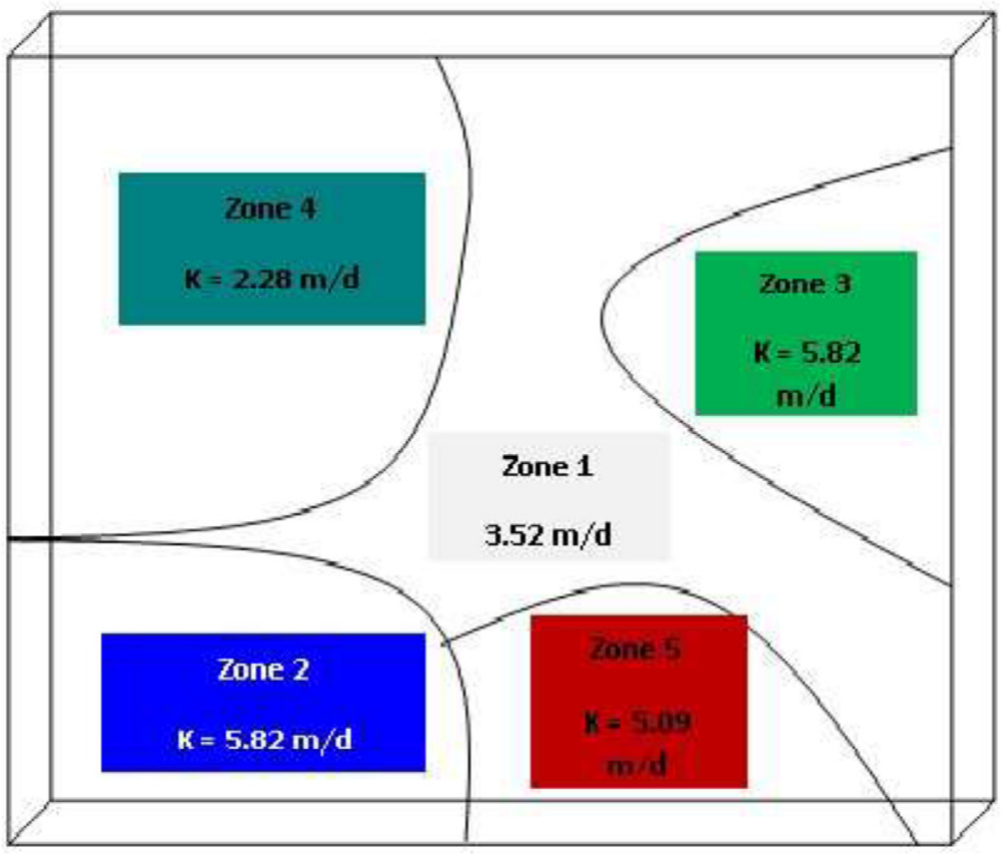
Hydraulic conductivities zones.

Static water levels records of the wells were used as initial heads. The initial heads were interpolated within the model to obtain the initial heads for the entire model (Fig. 1e). The observation heads were interpolated using the nearest Neighbour technique. Table 3 indicate the parameters used in the developed groundwater flow model.

**Fig. 1e:**
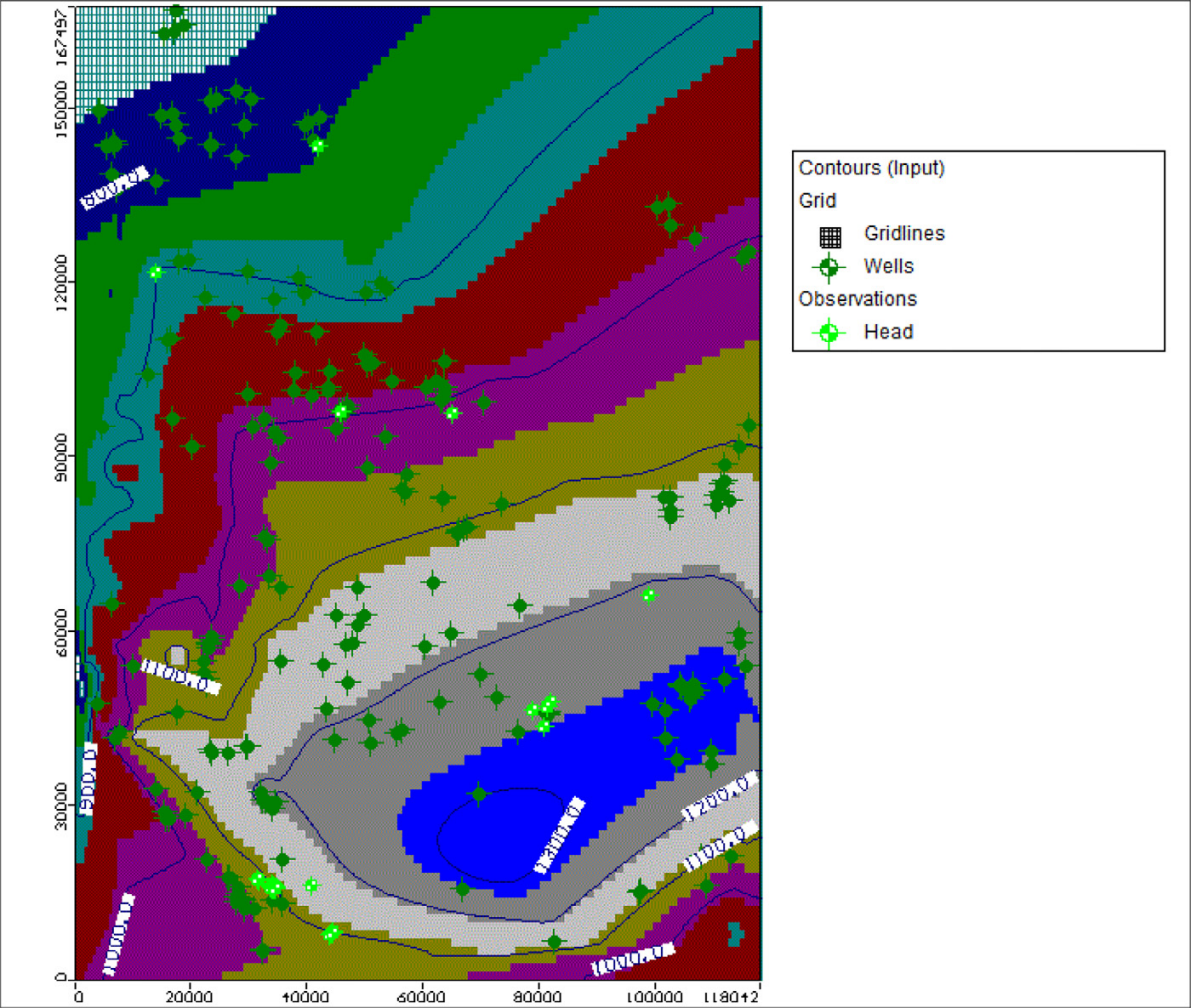
Initial heads with contours simulated for study area.

**Table 3:** Parameters value for model inputs.

Parameters	Range
Hydraulic conductivity (m/d); K_x_	0.003568 -30.78
Specific storage (m^−1^) S_s_	0.0015 – 0.00201
Specific yield S_y_	0.20
Effective porosity	0.15
Total porosity	0.30

*Boundary conditions*: Three types of boundary conditions were incorporated in this study which included; constant head, river and recharge. Dirichlet boundary conditions (fixed boundary condition) of the study area were used as a constant head. The constant head was allocated within a minimum distance of 2 km from pumping to boundary of the modelled aquifer. A boundary located at a distance of 2 km will ensure that effect of pumping from the nearest well will not be felt at such a distance.

*River:* MODFLOW simulated the interaction between the surface and groundwater via seepage layer separating the surface water from groundwater system. Four rivers were included in the model; Sandriver, Houtriver, Seepabana and Brak rivers (Table 4). All rivers allocated within the model domain were imported from ArcGIS as shapefiles. Stage elevation, river depth and thickness data were obtained from Department of Water Affair (DWA). River bottom elevations were obtained through deduction of river depth from stage elevation. The conductance was automatically calculated in MODFLOW using Eq. (1).

(1) $$C=\frac{K.L.W}{M}$$

**Table 4:** Rivers information included in MODFLOW model.

Name of rivers	River Stage elevation (m)	River Bottom elevation (m)	Riverbed Thickness (m)	Depth (m)
Sandriver	1196	1197.271	1.2	1.271
Brakriver	889	890.2	1.2	1.2
Houtriver	1362	1363.2	1.2	1.2
Seepabana	1107	1108.721	1.2	1.721

(1), (2)

*Where;* C is Conductance value, L is length of a reach through a cell, W is Width of the river in the cell, *M* is thickness of the riverbed, *K* is the vertical hydraulic conductivity of the riverbed material.

**Recharge Package (RCH):** The study area recharge polygon shape file map was imported in MODFLOW under boundaries. At this point recharge was considered as certainty parameter for deterministic model. Fig. 1f shows the boundary conditions used as input in to groundwater flow model (deterministic) where recharge is applicable.

**Fig. 1f:**
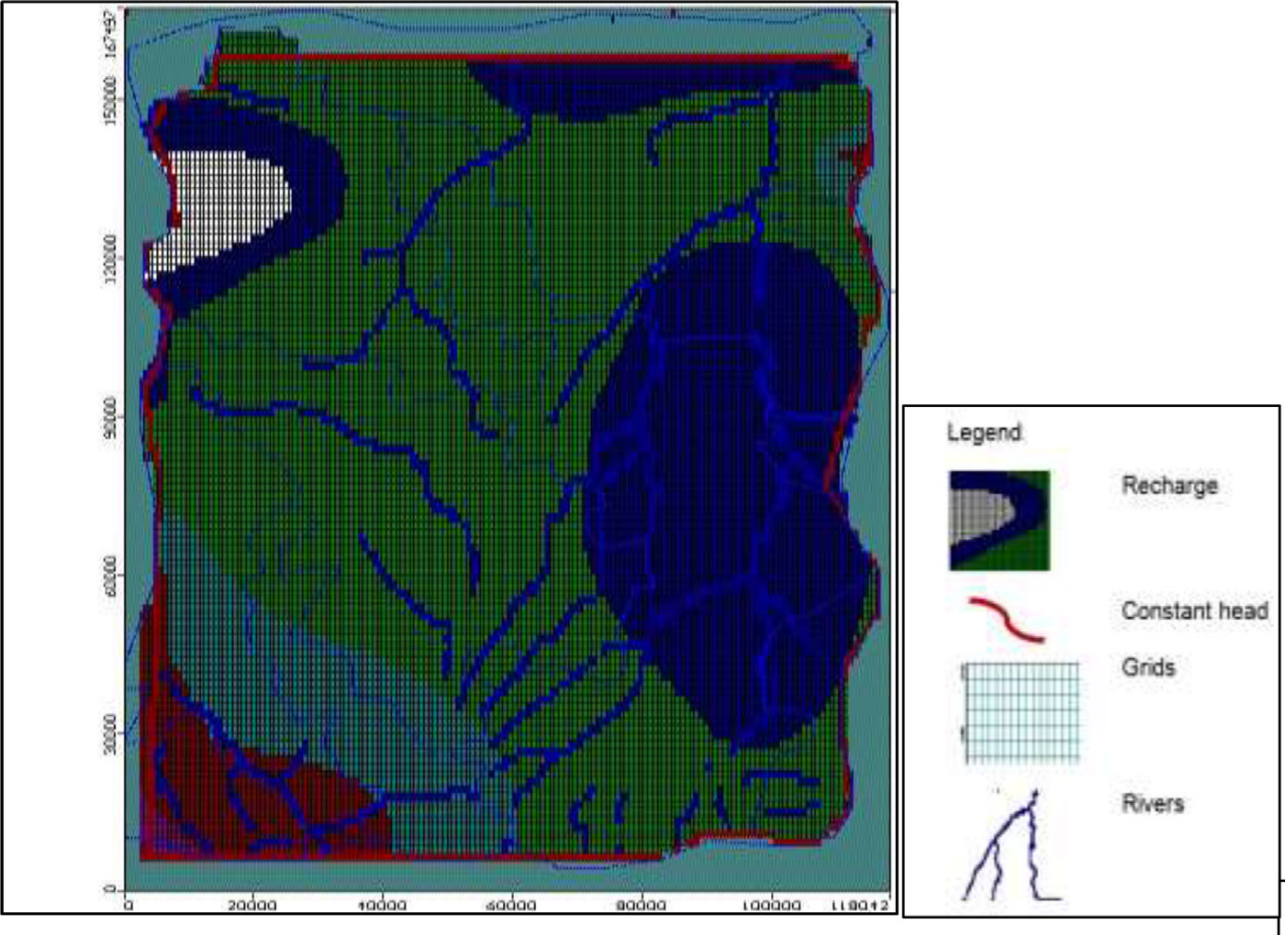
Section of model area showing boundary condition**s**.

### 1.2 Model simulation

After all the input were imported in the model, the model was then simulated using MODFLOW and Zone budget through the run engine (Fig. 1g). The simulated model aimed to produce results for zone budget and mass balance statistics. The model was run for one stress period for 360 days with maximum outer (MAXITER) iteration of 50 and maximum inner iterations (ITERI) of 25 (Fig. 1h). (This parameter provides an upper limit on the number of outer iterations to be performed. The maximum number of iterations will only be used if a convergent solution is not reached beforehand.)

**Fig. 1g:**
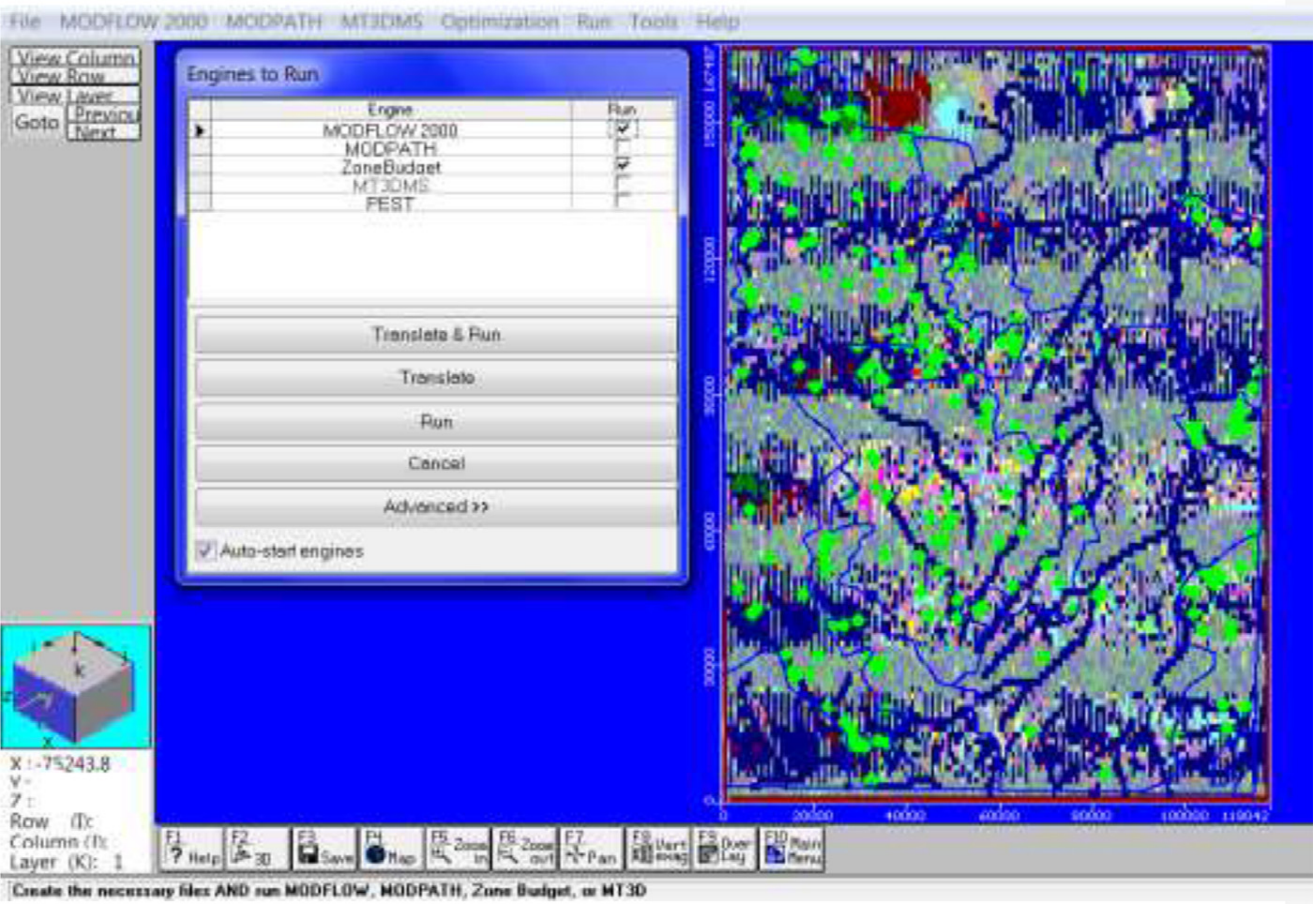
Display of Engine to run for MODFLOW and model inputs.

**Fig. 1h:**
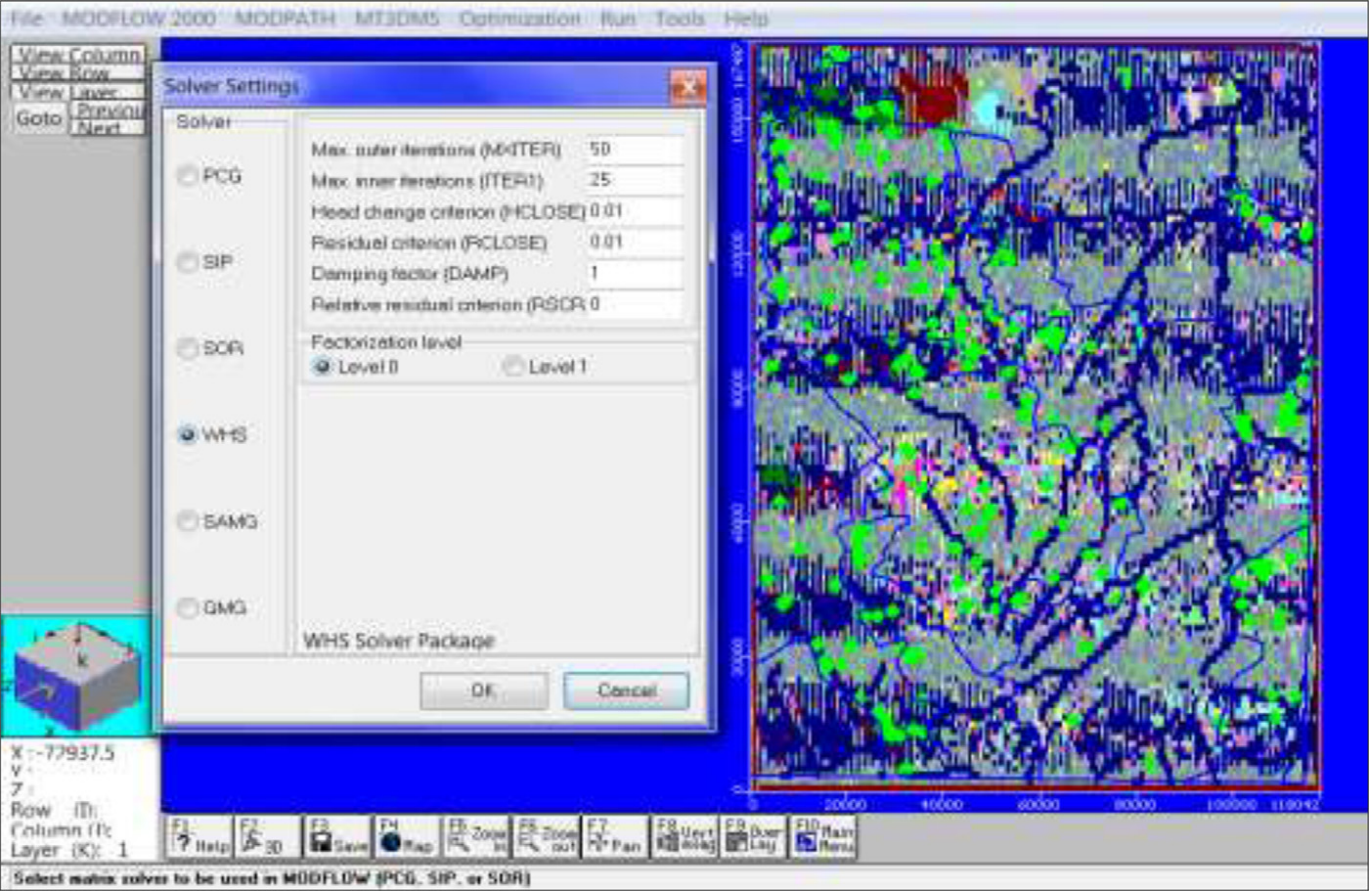
Display of MODFLOW Solver settings and model inputs.

### Step 2: Model Calibration

A total of 30 observation wells from GRIP database were selected for the calibration of steady state model. In this study, the hydraulic conductivity was used as parameter of interest for calibration. The calibration was done using PEST module (the parameter estimation model developed by [2] Doherty [2]) within MODFLOW. PEST applies the use of a nonlinear least-squares regression method for determination of model parameters while minimizing the following objective function (Eq. 2):

(2) $$\Phi =\;\sum_{i=1}^{N}{\left(w_{i}r_{i}\right)}^{2}$$

Where Φ, is the objective function which in reality is sum of the squared weighted residuals, *N* is the number of measurements *w_i_*, is the weight for the *i^th^* measured quantity, and *r_i_* is the *i^th^* residual (*i^th^* measured quantity – *i^th^* simulated quantity).

Five zones of hydraulic conductivities were used during the calibration process. PEST executed MODFLOW with the initial hydraulic conductivity's values of five zones and output of MODFLOW were compared with observed data. The evaluation of calibration was done by comparing predicted and observed heads through scatter plot and by applying the three common ways of error quantifying methods which are (i) Mean Error (ME) (ii) Mean absolute error (MAE) and (iii) Root Mean Square Error (RMSE) adopted from [1] Anderson and Woessner [1].

### Step 3: Model Validation

The model was validated using 30 boreholes (Table 5). In order to validate the model, dataset of 2011 boreholes were selected. This is because most of the data needed for the model was available in this year. The preparation of validation wells was done using Excel and ArcGIS and later on imported to MODFLOW using Well import tool. The model performance statistics were evaluated using ME, MAE, RMSE and Normalised Root Square Mean (NRMS).

**Table 5:** Dataset of 2011 well used for validation.

WELL_NAME	X [m]	Y [m]	Screen ID	SCREEN_ELE	Obs. Time [day]	HEAD [m]
VW1	701095	7334120	VW1	1111.28	365	1117.78
VW2	701679	7333460	VW2	1114.1	365	1124.1
VW3	701425	7333910	VW3	1115.67	365	1123.17
VW4	700755	7334390	VW4	1106.46	365	1118.96
VW5	699738	7335980	VW5	1111.13	365	1117.52
VW6	699392	7336130	VW6	1102.86	365	1111.76
VW7	699015	7336420	VW7	1101.54	365	1109.89
VW8	702462	7332400	VW8	1125.24	365	1129.38
VW9	702058	7333090	VW9	1120.84	365	1129.62
VW10	701957	7332940	VW10	1119.39	365	1125.34
VW11	701865	7332840	VW11	1115.71	365	1128.49
VW12	702211	7332590	VW12	1123.37	365	1132.65
VW13	701899	7332580	VW13	1115.19	365	1121.79
VW14	700549	7433910	VW14	915	365	922.8
VW15	681608	7438820	VW15	890	365	897.8
VW16	700523	7433970	VW16	919	365	927.4
VW17	713390	7414850	VW17	1015.70	365	1030.7
VW18	732503	7414420	VW18	1015	365	1022.8
VW19	749642	7356850	VW19	1252	365	1259.8
VW20	747207	7356060	VW20	1215	365	1222.8
VW21	750941	7350730	VW21	1255	365	1262.8
VW22	749616	7355880	VW22	1225	365	1232.8
VW23	749742	7352160	VW23	1250	365	1257.8
VW24	745289	7363540	VW24	1200	365	1207.8
VW25	749420	7364880	VW25	1180	365	1187.8
VW26	763775	7346010	VW26	1300	365	1307.8
VW27	772557	7369760	VW27	1175	365	1182.8
VW28	709298	7460550	VW28	835	365	842.8
VW29	706128	7326200	VW29	1146.46	365	1154.07
VW1	706681	7333200	VW30	1151.46	365	1152.87

In summary the first section of the methodology was development of groundwater flow model (which is deterministic in nature). Thus in order to develop stochastic model, recharge uncertain parameter was used as input in deterministic model. The methodology on the development of a stochastic solution is presented below;

PART 2: Methodology on the development of a stochastic solution

### 2.1 Problem formulation

This study considers the fact that uncertainty exists in groundwater recharge, an input parameter of groundwater flow modelling. The methodology considered the usual groundwater flow Eqn 3

(3) $$S_{s}=\frac{\partial h\;}{\partial t}=\frac{\partial }{\partial x\;}\left(K_{x}\frac{\partial h}{\partial x}\right)+\frac{\partial }{\partial y}\left(K_{y}\frac{\partial h}{\partial y}\right)+\frac{\partial }{\partial z}\left(K_{z}\frac{\partial h}{\partial z}\right)\pm w$$

Where: K_x_, K_y_, K_z_ are hydraulic conductivity along the x, y and z coordinate axes, which are assumed to be parallel to the major axes of hydraulic conductivity [ms^−1^]; h is the potentiometric head [m]; w: Volumetric flux per unit volume, representing sources and/or sinks of water [m^3^s^−1^]; Ss: the specific storage of the porous material [m^−1^]; t: time [s].

− w = discharge
+ w = recharge

Eq. (3) can be used to simulate the unknown variable (e.g. recharge). If the parameters in Eq. (3) are known with certainty, then the groundwater simulation is said to be deterministic, otherwise stochastic. If - w can be correctly estimated, it means Eq. (3) can be used to accurately determine the amount of groundwater storage.

However, Eq. (3) is a partial differential equation (complex) which cannot be solved analytically; hence we need to call upon numerical methods which will convert the partial differential equation to ordinary differential equation which is solvable through numerical methods. By disaggregating w term in Eq. (3) into -w and +w, Eq. (3) changes into the following Eqn 4:

(4) $$S_{s}=\frac{dh\;}{dt}=\frac{d}{dx\;}\left(K_{x}\frac{dh}{dx}\right)+\frac{d}{dy}\left(K_{y}\frac{dh}{dy}\right)+\frac{d}{dz}\left(K_{z}\frac{dh}{dz}\right)-w_{1}+w_{2}$$

From Eq. (4), the interest was to quantify *w_2_* (recharge) which is an input to groundwater model. In this case the quantification of recharge was based on spatial variability (x, y) and temporal variability (time dependent). This implies that, *w_2_* changes from being homogeneous to heterogeneous and dynamic Eqn 5.

(5) $$S_{s}=\frac{dh\;}{dt}=\frac{d}{dx\;}\left(K_{x}\frac{dh}{dx}\right)+\frac{d}{dy}\left(K_{y}\frac{dh}{dy}\right)+\frac{d}{dz}\left(K_{z}\frac{dh}{dz}\right)-w_{1}+w_{2\left(\left(x,y\right),t\right)}$$

Eq. (5) is deterministic in nature. However, since we appreciate the fact that recharge is not deterministic but depends on climate conditions (which are themselves uncertain), we need to regard it as uncertain and hence stochastic. In this study stochastic approach was used to solve the uncertainty problem. For instance, if we take recharge as uncertainty (µ); Eq. (5) becomes (1), (6).

(6) $$S_{s}=\frac{dh\;}{dt}=\frac{d}{dx\;}\left(K_{x}\frac{dh}{dx}\right)+\frac{d}{dy}\left(K_{y}\frac{dh}{dy}\right)+\frac{d}{dz}\left(K_{z}\frac{dh}{dz}\right)-w_{1}+w_{2\left(\left(x,y\right),t,\mu \right)}$$

This is now a fairly complex equation to solve because the problem is not only heterogeneous and dynamic, but also uncertain and hence stochastic.

### 2.2 Stochastic methodology procedure

Stochastic uncertainty analyses were performed by randomly sampling recharge realisation and running a series of Monte Carlo (MC) simulations. In this research, the random numbers were generated by using Pseudo-random number generators. The random numbers were generated using mean recharge value ($\bar{\mu }$) of 105 mm/yr and standard deviation (σ) of 58. MATLAB code was written to perform MC simulation based on Gaussian mixture models (GM) sampling method (for realization mapping) which uses expectation maximization (EM) algorithm implemented under MATLAB 2014a environment.

**MATLAB CODE**

%pd = makedist('Lognormal')

pd = makedist('lognormal','mu',105.00,'sigma',log(58.00));

Mm = mean(pd);

Mnv = Mm;

%The mean of the lognormal distribution is not equal to the mu parameter.

%Generate random numbers from the lognormal distribution and compute their log values.

rng(1); % for reproducibility

Y = random(pd,117,164,500);

X = log(Y);

R1 = X(:,:,1);

RN1 = R1(:);

R2 = X(:,:,2);

RN2 = R2(:);

R3 = X(:,:,3);

RN3 = R3(:);

R4 = X(:,:,4);

RN4 = R4(:);

R5 = X(:,:,5);

RN5 = R5(:);

R6 = X(:,:,6);

RN6 = R6(:);

R7 = X(:,:,7);

RN7 = R7(:);

R8 = X(:,:,8);

RN8 = R8(:);

R9 = X(:,:,9);

RN9 = R9(:);

R10 = X(:,:,10);

RN10 = R10(:);

Continues up to 1000……………. 1000 X (:,:,1000);

%Compute the mean of the logx values.

m = mean(logX); figure subplot(2,1,1) scatter(logX1,logX2,10,'ko') %scatter(logx,10,'ko') xlabel('logx') ylabel('logy') subplot(2,1,2) hist(logX,50) xlabel('logx') ylabel('logy')

figure scatter(logX1,logX2,12,'bo') xlabel('Recharge [mm/year]') ylabel('Recharge [mm/year]')

**Fig. 2a:**
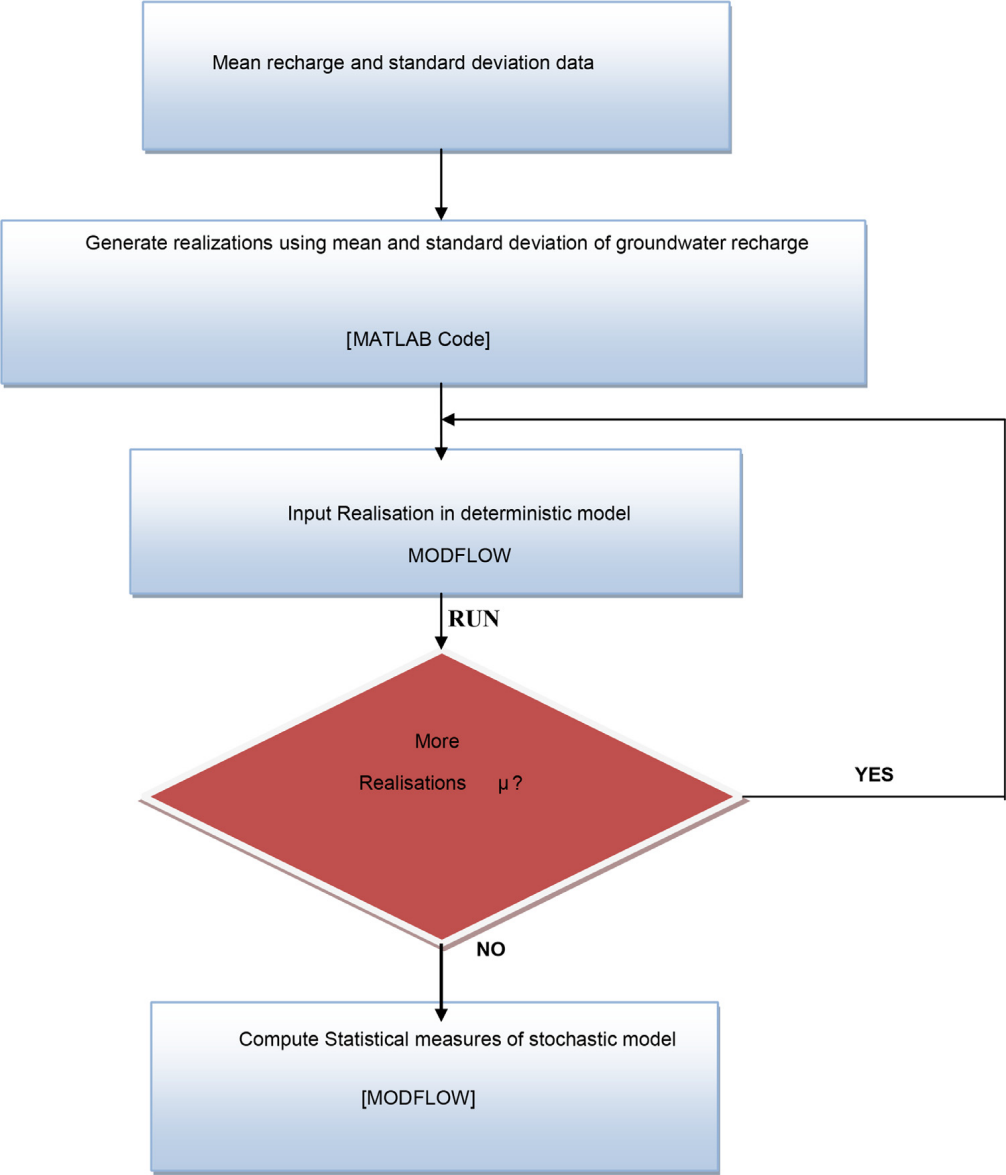
Flow chart for stochastic solution methodology.

Sets of random numbers generated from this distribution were then used in groundwater flow simulation model (deterministic) to determine simulated hydraulic heads. The procedure was repeated by generating more sets of possible outputs up to 1000 realizations. This process is a core procedure for Monte Carlo method. The randomly generated recharge grid cell was based on 117 × 164 rows and columns of the study area, resulting in 19188 model grid cells of recharge values. The expected mean recharge was based on 50, 100, 300, 500, 800 and 1000 realizations. A series of Monte Carlo (MC) experiments was done in order to determine the number of realisations for which the residual mean was insensitive to increasing number of realisations used. A Monte Carlo (MC) technique was used to solve stochastic groundwater problem. The flow chart of stochastic solution methodology is shown in Fig. 2a.

For purposes of clarity on how uncertainty was evaluated in the simulation model, (see Eq. 5), assume recharge is a random variable denoted by µ, hence, by considering the source/sink term in Eq. (5) as being stochastic, Eq. (5) can be transformed into Eq. (6) which becomes stochastic because its solution depends on the outcomes of realisations of the random values of recharge (This study dealt with two dimensional therefore the *K_z_* dimension falls off). Thus, a large number of realisations of recharge were generated and Monte Carlo (MC) approach was used to solve the problem repeatedly using methodological steps shown in Fig. 2a.

This study implies that the stochastic model can be used as an appropriate tool to manage the groundwater resources in the study area.

## CRediT authorship contribution statement

**Sophia Rwanga:** Conceptualization, Methodology, Software, Formal analysis, Investigation, Data curation, Writing - original draft, Visualization. **Julius Ndambuki:** Supervision, Data curation, Visualization, Writing - review & editing.

## Declaration of Competing Interests

The authors declare that they have no known competing financial interests or personal relationships that could have appeared to influence the work reported in this paper.
